# Ebola Viral Disease Outbreak-2014: Implications and Pitfalls

**DOI:** 10.3389/fpubh.2014.00263

**Published:** 2014-12-01

**Authors:** Mahip Acharya

**Affiliations:** ^1^Independent Researcher, Kathmandu, Nepal

**Keywords:** Ebola virus outbreak, zoonosis, pharmaceutical industries, ethics in drug approval, global health

West Africa – consisting of countries such as Guinea, Liberia, Sierra Leone, and Nigeria – is the westernmost region of the African continent. Afflicted by widespread poverty and political instability, West Africa is one of the poorest regions in the world – Liberia, Sierra Leone, and Guinea are respectively ranked 174, 177, and178 out of 187 countries on the United Nations Development Program Human Development Index (1). This region is in the news of late, not because of the poverty *per se* but due to one of its repercussions – its impact on health. The West African country Guinea encountered a new epidemic in March this year, which subsequently encroached upon Liberia and Sierra Leone and turned out to be an unprecedented event in health sector of West Africa (2). Ebola, a viral disease characterized by fever, diarrhea, vomiting, and hemorrhage (3), entered this region and started a disease outbreak. So great has been the havoc of this outbreak that 3685 cases and 1841 deaths have been reported as of 31 August 2014 (4). These are mere tangible effects of the disease; it has stirred a monumental fear in people around the globe, to say nothing of people from these suffering countries.

Ebola virus is a filamentous virus, five species of which have been identified; three of these species have been responsible for outbreaks in sub-Saharan African countries such as Sudan, Republic of Congo, Gabon, and Uganda (3). Zaire ebolavirus, also known as EBOV, is responsible for the ongoing Ebola hemorrhagic fever outbreak; its clinical manifestations closely resembles Marburg viral disease, a disease native to Africa and caused by another filovirus named Marburg virus (3). Its first appearance – and detection – harkens back to 1976, when it was observed during a hemorrhagic fever outbreak in southern Sudan and northern Zaire (5). Ebola viral disease, a zoonotic disease, has since been observed intermittently in different regions of Central Africa (6), but none of those has wreaked damage as monumental as the ongoing outbreak.

The current Ebola viral disease outbreak is turning out to be an insidious threat to the public health of West Africa – and by extension, to global health. Widespread poverty and corruption, underdeveloped health care systems, and lack of proper education in these West African countries have been a significant factor in the eruption and spread of this disease. Due to these and other reasons, most of these African nations have been unable to stem the outbreak. One exception to this has been Nigeria, which has been able to control this epidemic (7). A lot of effort has been put into reversing this epidemic, primarily from developed nations and international organizations. The outbreak, however, still remains to be brought under control. This has raised issues regarding our health systems, state-of-the-art research facilities, and regulatory laws pertaining to health. This outbreak stands out as a health emergency and also displays some of our collective limitations.

## Implications of the Outbreak

The ongoing outbreak has raised concerns about the flexibility and timeliness of research and development of drugs and vaccines. We have come a long way in regards to technological development and innovation, especially in the last few decades. Yet, an efficacious treatment or prevention for Ebola could not be made available in the hour of need, despite vigorous research efforts. Existence of numerous strains of a single virus species and their continued mutation – as is the case with the current Ebola infection (8) – often stymies the research for anti-viral drugs. This is similar to the case with antibiotics, where bacterial resistance is a chief concern (9). Bacterial and viral diseases benefit from the ever-evolving genetic makeup of their agents, which are capable of rapidly rendering a treatment ineffective. Despite advances in genome sequencing (10), the current Ebola outbreak has reinforced the notion that research in therapeutics, especially for viral and bacterial infections, has a long way to go.

Diseases such as Ebola fall in the category of rare diseases, which do not contribute significantly to disease burden in the affected areas, let alone globally. That Ebola, first identified in 1976, has occurred sporadically in the last three decades does not encourage the discovery of drugs for the disease. Far fewer people have been affected by Ebola as compared to some global diseases such as tuberculosis or malaria (11). The lack of incentives for treatments of such intermittently occurring diseases, rather than the inability of pharmaceutical industries, has been pointed out to be the reason for non-existence of efficacious treatments for the disease (12). There is not much monetary benefit for pharmaceutical industries in this line of research. As a result, pharmaceutical industries are not foraying into research for therapeutics for such rare diseases (11, 12). This issue has been raised earlier with respect to the unwillingness of pharma industries to get involved in antibiotics research (13). The culpability of pharma industries, however, is not as black and white as it is claimed to be. Innumerable rare diseases are prevalent throughout the world, and taking initiatives for developing drugs for each of these diseases is not always possible or financially advisable. On the other hand, neglecting these diseases could lead to serious consequences, as indicated by current Ebola outbreak. The current outbreak has fueled the criticism of pharmaceutical industries for being market-driven. More significantly, it has highlighted the predicament of health and drug research domain.

The economic and political implications of the current outbreak have grabbed more attention. The fact that the outbreak has occurred in West Africa and that these regions possess threadbare socio-economic framework, highlights the relationship of weak economic and political systems with the ongoing epidemic. Guinea, Liberia, and Sierra Leone – the epicenter of the current Ebola outbreak – were at the receiving end of civil conflicts (in Liberia and Sierra Leone) and an inefficient government (in Guinea) for decades. This has left these countries with debilitated infrastructures, health sector being no exception (1, 2). Political upheavals indeed disrupt the very fabric that holds a country together, and this disruption has spilled over to the economic and health sectors in these African countries. That Nigeria, a comparatively developed and more stable West African country, has been able to check the spread of Ebola bolsters the importance of political and economic stability (7). Moreover, poverty is deep-rooted, and resources are limited in these countries. In fact, poverty has been regarded as the single most important factor for the unbridled nature of this outbreak (14). Admittedly, the current Ebola outbreak calls for a closer look at the socio-economic and political background of countries fraught with epidemics.

This outbreak has also brought to the fore the significance of education, environmental factors, and cultural factors in a disease outburst. Ebola, being a zoonotic disease, has afflicted humans owing to greater reliance of people on animal products, predominantly meat, and repeated visits to forests and mines for wood and minerals (1). Traditional practices in disease-affected West African countries are regarded to be health hazard-prone (bathing of corpses by hand before burial, for instance), which have been instrumental in perpetuating the outbreak (2). Further, the lack of proper education in the people and their unwillingness to accept the existence itself of the disease have been major threats in checking its spread.

The current outbreak has underscored a certain flaw in handling ethical issues at the time of emergency. The ethical requirements surrounding the approval of new drugs have come under scrutiny, courtesy of the current outbreak. Drugs that have shown positive effects against Ebola virus in laboratories and on animal subjects are waiting for their use in Ebola patients – this long wait has resulted from rigorous requirements of safety and efficacy testing in human subjects. One such product is ZMapp – a combination of three monoclonal antibodies – which has shown successful abortion of Ebola infection in non-human primates (monkeys) (15). It has been successfully employed in the treatment of two United States healthcare workers, the therapy being driven by compassionate use rather than substantial evidence – efficacy and safety testing have not been carried out on human subjects (16). With limited data about efficacy and toxicity of the therapy, its effect on treating Ebola infection has not been well-established. This raises an important question about design of clinical trials and its modification based on urgent needs. Changes in the current lengthy procedure for drug approval to allow for rapid testing and acceptance of therapies, especially in critical times, are considered to be a remedy for such bottlenecks (17). Clearly, the *status quo* of drug testing, approving, and regulating does not hold good in certain cases of disease outburst, thus warranting a tweaking of presently established guidelines. The current outbreak has been instrumental in underlining the significance of the regulatory aspect of new therapies.

## Pitfalls

The ongoing Ebola outbreak raises some serious questions, the answers to which could be harrowing. What if this outbreak or such types of viral outbreaks happen not to be rare events? What if Ebola and Marburg are just the tip of the viral iceberg and plenty more are bracing themselves for human infection? Although these questions sound cynical, it is high time that these questions were asked. In fact, concerns were raised earlier about the negligence of Ebola outbreaks in the past, with warnings that a bigger outbreak could arise (18). We are not well-informed about the entire arsenal of microbes in nature, as this outburst of a zoonotic disease demonstrates. Moreover, urbanization and globalization are in full swing, and have forced people to exploit the nature for sustenance (18). With greater delving into the nature for resources, there is enhanced possibility of transmission of newer and possibly more threatening diseases. We have already been grappling with HIV, which is a global health issue at the moment. This outbreak could well point to a future marred with such other health threats, if serious deliberations are not performed by professionals of different backgrounds, including economists, political leaders, and public health experts.

The current Ebola spread, which could have been limited to few people and to a small area through public health measures alone, has snowballed into widespread havoc. Non-pharmaceutical interventions have been shown to be effective in curtailing the spread of a disease, as observed with Influenza pandemic in the United States (19). In order to implement public health measures, however, the health sector needs to be strong, which is not the case in African countries. Diseases such as Ebola exist at the intersection of environmental, socio-economic, cultural, and public health systems of a society. Eruption of such a disease indicates serious flaws in more than one system, which is evident in the ongoing Ebola outbreak.

In conclusion, a consensual effort on the part of local and international health bodies, pharmaceutical industries, and public health experts may, hopefully, end the current outbreak soon. In doing so, the inherent message of this outbreak needs to be assimilated as well, and measures ought to be taken to prevent future outbreaks. Surely, we do not want to make an HIV/AIDS, a global health threat, out of other such infectious diseases.

## Conflict of Interest Statement

The author declares that the research was conducted in the absence of any commercial or financial relationships that could beconstrued as a potential conflict of interest.
